# Molecular prevalence of intestinal parasites infections in children with diarrhea in Franceville, Southeast of Gabon

**DOI:** 10.1186/s12879-020-05071-x

**Published:** 2020-05-15

**Authors:** Sandrine Lydie Oyegue-Liabagui, Nal Kennedy Ndjangangoye, Lady Charlene Kouna, Gwladys Mirlande Lekolo, Franck Mounioko, Sylvie Kwedi Nolna, Jean Bernard Lekana-Douki

**Affiliations:** 1 Ecole Doctorale Régionale d’Afrique Centrale en Infectiologie Tropicale (ECODRAC), Université des Sciences et Techniques de Masuku, BP 876 Franceville, Gabon; 2 Unité d’Evolution, Epidémiologie et Résistance Parasitaire (UNEEREP), Centre International de Recherches Médicales de Franceville (CIRMF), BP 769 Franceville, Gabon; 3 Laboratoire d’Ecologie Vectoriel, Institut de Recherche en Ecologie Tropicale, BP 13354 Libreville, Gabon; 4 Capacity for Leadership Excellence And Research (CLEAR, Inc.), Yaoundé, Cameroon; 5 Département de Parasitologie-Mycologie Médecine Tropicale, Faculté de Médecine, Université des Sciences de la Santé, BP 4009 Libreville, Gabon

## Abstract

**Background:**

Pediatric diarrhea caused by a range of pathogens, including intestinal parasites, is one of main causes of death among children under 5 years of age. The distribution of these parasitic infections overlaps in many environmental, socioeconomic and epidemiological settings. Their distribution and prevalence varies from region to region. In the current study, we assess the prevalence of intestinal parasites among pediatric patients with syndromic diarrheal disease living in Franceville, Gabon.

**Methods:**

A cross-sectional study conducted in the Amissa Bongo Regional Hospital and Chinese-Gabonese Friendship Hospital in Franceville, between November 2016 and August 2017, enrolled a total of 100 diarrheic children between 0 and 180 months of age. Parasite detection in stool samples was performed using molecular diagnostic by PCR. Difference in means were tested by Student’s t test and ANOVA while principal component analysis was used to determine the correlation between parasite distributions and age groups.

**Results:**

The overall prevalence of intestinal parasite infection was 61% (61/100). *Hymenolepis sp* and *Cryptosporidium hominis/parvum* were the most common parasites (31 and 19%, respectively), followed by *Encephalitozoon intestinalis* (15%), *Trichuris trichiura* (4%), *Dientamoeba fragilis* (4%), and *Enterocytozoon bieneusi* (2%). The polyparasitism rate was 19.7%, with 83.3% double and 16.7% triple infections. Protozoan infections (66.7%) were more prevalent than helminths infections (33.3%). Seasonal association of the circulation of intestinal parasite was statistically significant (*p* = 0.03). Correlations between different parasites was also observed.

**Conclusion:**

The prevalence of intestinal parasitic infections is highest in diarrheic pediatric children. The prevalence of parasitic infections indicates that protozoa and helminths are the most common parasites in the Franceville environment. This study reinforces the importance of routine examination of diarrheic stool samples for the diagnostic of intestinal parasites. Further analyses are required to better understand the local epidemiology and risk factors associated with the transmission of intestinal parasites in Franceville, Gabon.

**Keyswords:**

diarrhea, children, intestinal parasitic infections, molecular diagnostic, Franceville, Gabon.

## Background

The World Health Organization (WHO) and United Nations International Children Emergency Fund define diarrhea as more than three loose or watery stools during a 24 h period. A duration of 14 days is the proposed criterion for acute diarrhea or persistent diarrhea [1]. According to the WHO, diarrheal disease is the world’s second leading cause of pediatric deaths, killing around 525,000 children under 5 years old annually. Globally, there are approximately 1.7 billion cases of childhood diarrheal disease every year and diarrhea due to infection is widespread throughout the developing world [2, 3]. In low-income countries, many of the risk factors for contracting diarrheal illnesses are linked to poor socioeconomic conditions, such as lack of access to safe water and sanitation, poor hygiene practices and unsafe human waste disposal [4, 5]. Diarrhea can be related to a wide range of bacteria, viruses, and intestinal parasites [6].

Intestinal parasitic infections (IPIs), such as soil-transmitted helminths (STHs) and parasitic intestinal protozoa, have been described as the major worldwide causes of illnesses and disease in tropical and sub-tropical regions in the world [3, 7]. Besides causing mortality and morbidity, IPIs have been associated with impairment of physical and intellectual development as well as worsening of nutritional status during infancy [6, 8, 9]. Indeed, they cause undernutrition, abdominal pain, diarrhea, intestinal obstruction, anemia, ulcers and other health problems that can lead to delayed cognitive development and impaired learning [10].

STHs are among the most common infections worldwide. More than 1.5 billion people, or 24% of the world’s population, are infected with soil-transmitted helminth infections worldwide. In areas where these parasites are intensively transmitted, over 267 million preschool-age children and over 568 million school-age children are infected [11]. Globally, 438.9 million people were infected with hookworm while 819 million were infected with *Ascaris lumbricoides*, and 464.6 million with *Trichuris trichiura*, with a large proportion occurring in Asia [3].

Intestinal protozoa that are most commonly associated with diarrhea in children include *Entamoeba histolytica, Giardia lamblia/duodenalis, Blastocystis sp, Cryptosporidium sp*, and *Dientamoeba fragilis* [12]. Globally, *Cryptosporidium sp* has been identified as the most common diarrhea-causing protozoan [13] and giardiasis is experienced by approximately 33% of people in developing countries [14, 15]. Like *Cryptosporidium sp* and *Giardia,* intestinal protozoa *Blastocystis sp* and *D. fragilis* are both relevant due to their significant public health and socioeconomic implications. Indeed, *D. fragilis* is frequently detected in co-infection with multiple protozoans, especially *Blastocystis sp* [16].

Depending on environmental, socioeconomic and geographical factors, the distribution and prevalence of IPIs varies from region to region. Gabon is a sub-Saharan African country with a high prevalence of intestinal infections [17, 18]. In a number of studies conducted in Gabon, the burden of IPIs was found to be high in urban and rural areas. Nationwide, the prevalence of IPIs was found to be 61.1%, including intestinal protozoa (56.7%) and soil-transmitted infections (22.2%). Factors such as inadequate water supplies, lack of proper health education and unsuitable sanitation favor the transmission and wide distribution of IPIs [19]. The distribution of intestinal parasites has been reported in some regions of Gabon, but few studies have assessed the prevalence of these parasites in pediatric diarrhea patients. A study conducted in Franceville on the etiology of diarrhea in children under 5 years old showed that viruses were the main agents responsible for diarrhea in children with an overall prevalence of 60.9% [20]. Another study conducted in Libreville, the capital of Gabon, showed that bacteria were responsible for acute diarrhea with a prevalence of 13% [21]. However, data on the involvement of IPIs in the etiology of infantile diarrhea in Franceville are not available. Conventional diagnostic of these intestinal parasites relies widely on microscopic examination of stool samples. However, coproscopic identification is limited by low sensitivity as evidenced in studies reporting a marked difference in the prevalence of intestinal parasites when comparing the results of coproscopic and molecular analyses [22]. The main objective of this study was to assess the prevalence of eighteen IPIs by microscopic and molecular diagnosis and their distribution according to age and seasonality, in children with diarrhea living in Franceville, south-east of Gabon.

## Methods

### Study area and participants

This study was conducted at the Amissa Bongo Regional Hospital and Chinese-Gabonese Friendship Hospital in Franceville, the capital of the Haut-Ogooué Province, Gabon. Children aged from 0 to 180 months old presenting a diarrhea syndrome with at least three bowel movements over 24 h were enrolled. Samples were collected between November 2016 and August 2017 in the pediatric wards of both hospitals and two samples, among which one blood sample and one stool sample, were collected per child. Over the weekend, stool samples were collected from children in the emergency ward. Samples were collected at home for children who were not admitted and left the hospital. After the inclusion of a patient, a clinical examination was conducted which was comprised of an interrogation, search of general signs, anthropometric parameters, and signs of dehydration. An examination of the digestive system was also performed. A brief clinical examination of the cardiopulmonary and neurological apparatus was also carried out.

### Sample collection

A total of two types of samples (whole blood and stool) were collected from the 100 children included. Blood (2 mL) was collected and distributed in EDTA tubes for a blood count test and in heparin tubes for urea, creatinine and ionogramm tests. When possible, stool samples were collected in sterile containers from diarrheal pediatric patients. Otherwise, a sterile container was given to the parents to collect the stool sample at home. These samples were analyzed by a direct examination followed by molecular analyzes. DNA was extracted using the QIAamp DNA Stool Mini kit (Qiagen, Hilden, Germany) according to the manufacturer’s instructions.

### Direct examination

Macroscopic examination was performed on each stool sample to note the appearance, consistency, color and possible presence of blood, mucus and adult forms of parasites. Direct microscopic examination after staining was carried out using the Kop-Color kit, according to the manufacturer’s instructions (Para-Selles/Kop-Color II Fumouze®. Fumouze Diagnostics; Levallois-Perret, France). The Kop-Color II kit is a differential staining process of parasitic elements using a mixture of staining agents one of which is Lugol. Briefly, after homogenizing the stool, a volume of stool equivalent to the size of a pea was placed in a hemolysis tube containing 1 mL of diluent (physiological water). The mixture is triturated and stirred to obtain a homogeneous suspension. 10 μL of KOP-COLOR II were placed on a slide and 1 drop or 25 μL of the stools suspension was added. The mixture thus obtained was well mixed. A coverglass was placed over the stool suspension and then examined using a microscope with white light (blue filter). Parasitic elements appear in yellow, yellow-orange or brownish-yellow on a more or less dark blue background.

### PCR detection

Eighteen [18] parasites, *Cryptosporidium hominis/parvum, Cystoisospora belli, Entamoeba hartmani, Entamoeba histolytica/dispar, Dientamoeba fragilis, Trichomonas intestinalis, Blastocystis hominis Enterocytozoon bieneusi, Encephalitozoon intestinalis, Ancylostoma duodenale, Necator americanus, Ascaris lumbricoïdes, Enterobius vermicularis, Strongyloïdes stercoralis, Trichuris trichiura, Schistosoma mansoni, intercalatum, haematobium, Hymenolepis sp*, were investigated using t sets of primers listed in Table 1. The identification of parasitic species was carried out using conventional PCR or multiplex amplification. Briefly, five microliters of DNA were amplified with a 1X Taq polymerase buffer (Invitrogen. Alphalab; Saint Laurent d’Aigouze, France), 0.8 μM of each primer, 0.2 mM dNTP (Invitrogen. Alphalab; Saint Laurent d’Aigouze, France), 1.5 mM MgCl_2_ and 0.024 U of Taq DNA polymerase (Invitrogen. Alphalab; Saint Laurent d’Aigouze, France), for *Cryptosporidium hominis/parvum, Cystoisospora belli, Entamoeba hartmani, Entamoeba histolytica/dispar, Dientamoeba fragilis, Trichomonas intestinalis, Blastocystis hominis Enterocytozoon bieneusi, Encephalitozoon intestinalis, Ancylostoma duodenale, Necator americanus, Enterobius vermicularis, Strongyloïdes stercoralis, Trichuris trichiura, Schistosoma mansoni, intercalatum, haematobium, Hymenolepis sp*. For *Ascaris lumbricoïdes*, five microliters of DNA were amplified with a 1X Taq polymerase buffer, 0.2 μM of each primer, 0.2 mM dNTP, 1.5 mM MgCl2 and 0.024 U of Taq DNA polymerase. Specific cycling programs for each species are described in the corresponding references mentioned in Table 1. PCR products were detected by 2% agarose gel electrophoresis stained with GelRed® (Interchim; Montluçon, France).

**Table 1:** Sequences of primers sets used and targeted genes

Intestinal parasite	Names	Primers (5′ → 3′)	Target region	Reference
**Protozoa**				
*Cryptosporidium parvum/hominis*	1PSF	AAC TTT AGC TCC AGT TGA GAA AGT ACT C	hsp70 gene	(Garces-Sanchez, Wilderer et al. 2009)
	1PSR	CAT GGC TCT TTA CCG TTA AAG AAT TCC		
*Blastocystis hominis*	BH1	GCT TAT CTG GTT GAT CCT GCC AGT	16 like RNA	(Init, Foead et al. 2007)
	BH2	TGA TCC TTC CGC AGG TTC ACC TAC A		
*Cystoisospora belli*	Ib213F	GGA TAT TCC CTG CAG CAT GT	5,8S rRNA/ITS2	(Taniuchi, Verweij et al. 2011)
	Ib213R	CGG GAC ACA ACT CAA CAC TG		
*Encephalitozoon intestinalis*	Eint214F	CAC CAG GTT GAT TCT GCC TGA C	SSU rRNA	(Verweij, Ten Hove et al. 2007)
	Eint214R	CTA GTT AGG CCA TTA CCC TAA CTA CCA		
*Enterocytozoon bieneusi*	EbITS-89F	TGT GTA GGC GTG AGA GTG TAT CTG	SSU rRNA	(Verweij, Ten Hove et al. 2007)
	EbITS-191R	CAT CCA ACC ATC ACG TAC CAA TC		
*Dientamoeba fragilis*	DFpn_1f	GCC AAG GAA GCA CAC TAT GG	SSU rRNA	(Roser, Nejsum et al. 2013)
	DFpn_364r	GTA AGT TTC GCG CCT GCT		
*Trichomonas intestinalis*	TH3	TGT AAA CGA TGC CGA CAG AG	SSU rRNA	(Crucitti, Abdellati et al. 2004)
	TH5	CAA CAC TGA AGC CAA TGC GAG C		
*Entamoeba histolytica*	EnthF	ATG GCC AAT TCA TTC AAT GA	SSU rRNA	(Suzuki, Kobayashi et al. 2008)
	EnthR	TAC TTA CAT AAA GTC TTC AAA ATG T		
*Entamoeba dispar*	EntdF	GTT AGT TAT CTA ATT TCG ATT AGA AC	SSU rRNA	(Suzuki, Kobayashi et al. 2008)
	EntdR	ACA CCA CTT ACT ATC CCT ACC TA		
*Entamoeba hartmanni*	EhartF	GTG AAG AGA AAG GAT ATC CAA AGT	SSU rRNA	(Suzuki, Kobayashi et al. 2008)
	EhartR	ATA TCA TTT TCA ACT ACG AGC		
**Helminths**				
*Ascaris lumbricoides*	Alum96F	GTA ATA GCA GTC GGC GGT TTC TT	ITS1	(Wiria, Prasetyani et al. 2010)
	Alum183R	GCC CAA CAT GCC ACC TAT TC		
*Enterobius vermicularis*	EVpn_1f	CAA CAC TTG CAC GTC TCT TCA	5S rRNA	(Roser, Nejsum et al. 2013)
	EVpn_195r	ATT GCT CGT TTG CCG ATT AT		
*Trichuris trichiura*	TtF	TTG AAA CGA CTT GCT CAT CAA CTT	18S	(Liu, Gratz et al. 2013)
	TtR	CTG ATT CTC CGT TAA CCG TTG TC		
*Strongyloides stercoralis*	Stro-1530F	GAA TTC CAA GTA AAC GTA AGT CAT TAG C	18S	(Verweij, Canales et al. 2009)
	Stro-1630R	TGC CTC TGG ATA TTG CTC AGT TC		
*Ancylostoma duodenalis*	Ad125F	GAA TGA CAG CAA ACT CGT TGT TG	ITS2	(Verweij, Brienen et al. 2007)
	Ad195R	ATA CTA GCC ACT GCC GAA ACG T		
*Necator americanus*	Na58F	CTG TTT GTC GAA CGG TAC TTG C	ITS2	(Verweij, Brienen et al. 2007)
	Na158R	ATA ACA GCG TGC ACA TGT TGC		
*Schistosoma mansoni*	Smcyt748F	CCC TGC CAA ATG AAG AGA AAA C	Mitochondrial DNA	(ten Hove, Verweij et al. 2008)
	Smcyt847R	TGG GTG TGG AAT TGG TTG AAC		
*Schistosoma intercalatum*	Sh110	TTC CTC CAA CTA CCA TCT TAT CTC	Sh110	(Abbasi, King et al. 2007)
	Sm-SL	AAC CGT CAC GGT TTT ACT CTT GTG		
*Hymenolepsis sp.*	Pr-a	TGG TTT TTT GTG CAT CCT GAG GTT TA	COI	(Muehlenbachs, Bhatnagar et al. 2015)
	Pr-b	AGA AAG AAC GTA ATG AAA ATG AGC AAC		

### Statistical analysis

The data collected were entered into an Excel spreadsheet and analyzed using the software package IBM SPSS Statistics version 21.0 (SPSS inc., Chicago, USA) and R software version 3.5.3. The Pearson Chi square for categorical variables, Student’s *t* test and ANOVA for the comparison of group means, were used. To compare multiple groups of data, the non-parametric Kruskal-Wallis test was used. The Mann-Whitney U non-parametric test was used for pairwise comparisons. Possible correlations between parasite distributions and age groups were identified using principal component analysis (PCA). The statistical significance was set at *p* < 0.05.

## Results

### Study population

A total of 100 diarrheal pediatric children aged from 2 to 169 months were included in the study. The sex ratio was 1.5 (60 males and 40 females). The mean and median age of children included were 24.8 and 14.5 months old, respectively. Diarrheal syndrome was predominantly found in children aged less than 48 months old (*p* < 0.005). The children’s demographical and clinical characteristics are summarized in Table 2. The mean age of the infected and uninfected children was 28.3 ± 4.3 and 19.4 ± 3.4 months old, respectively (no statistically significant difference). The differences were not statistically significant for body temperature and weight between infected (37.9 ± 0.2 °C and 11.9 ± 0.9 Kg; *p* = 0.5) and uninfected (38.2 ± 0.2 °C and 10.1 ± 1.05 Kg; *p* = 0.3) children. Haemoglobin, red blood cell, white blood cell and platelet values showed no significant difference between the two groups of children. Infected children had a significantly higher creatinine level (47.7 ± 3.8 mmol/L) than uninfected children (25.3 ± 2.7 mmol/L; *p* = 0.02).

**Table 2:** Demographical and hematological parameters of the patients (mean ± SD)

	Franceville children		
	Infected (*n* = 61)	Uninfected (*n* = 39)	p
Age (months)	28.3 ± 4.3	19.4 ± 3.4	0.06
Temperature	37.9 ± 0.2	38.2 ± 0.2	0.5
Weight (Kg)	11.9 ± 0.9	10.1 ± 1.05	0.3
Haemoglobin (g/dL)	10.4 ± 0.6	11.7 ± 0.5	0.1
Red blood cells (10^6^ cells/mm^3^)	4.3 ± 0.7	4.3 ± 0.2	0.7
White blood cells (10^3^ cells/mm^3^)	13.2 ± 2.1	9.7 ± 1.1	0.1
Platelets (10^3^ cells/mm^3^)	358.8 ± 313.8	361 ± 467.2	0.2
Creatinine (mmol/L)	47.7 ± 3.8	25.3 ± 2.7	**0.02**

leucocyte counts, hemoglobin concentrations and creatinine infected and uninfected children. Statistical significance was calculated using a student’s *t* test. SD = standard deviation

Clinical examination revealed that the most frequent signs associated with diarrhea were fever (60%; *n* = 60/100), vomiting (47%; *n* = 47/100), dehydration (17%; *n* = 17/100), weight-loss delay (16%; *n* = 16/100) and abdominal pain (8%; *n* = 8/100). These signs were assessed according to the usual clinical criteria: weight-to-height and height-to-age ratio, vomiting and fever.

### Prevalence of intestinal parasites

**Fig. 1:**
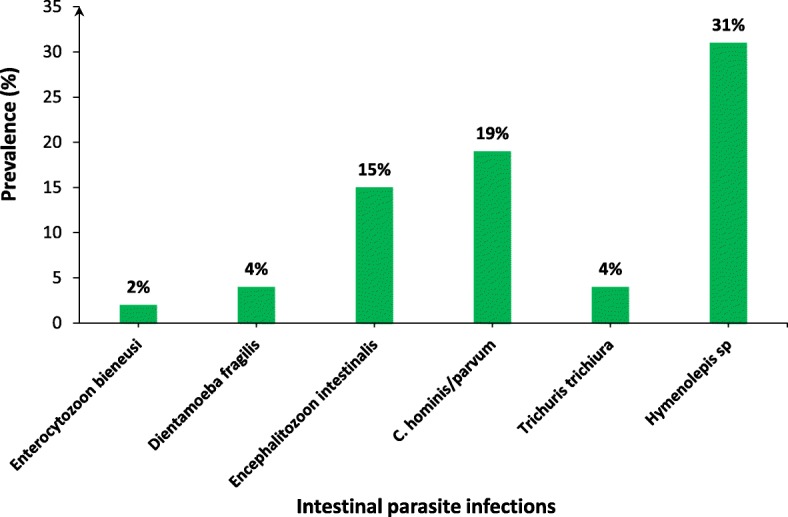
Prevalence of intestinal parasite infections. Parasite infection in 100 stools from these children were assayed using PCR. The number of positive cases are indicated on the figure, each bar represents the prevalence of each parasite

Microscopic examination of stool samples did not lead to the detection of parasites among the 81 participants examined. However, the PCR test revealed at least one intestinal parasite in 61 stools samples with 61% of global prevalence. There were six species of intestinal parasites among which four protozoans (*Dientamoebla fragilis, Encephalitozoon intestinalis, Enterocytozoon bieneusi, Crytosporidium hominis/parvum* and two helminths (*Trichuris trichiura and Hymenolepis sp*). *Cryptosporidium hominis/parvum* (19%) was the most prevalent protozoa, followed by *Encephalitozoon intestinalis* (15%). In helminths infections, the highest prevalence was observed with *Hymenolepis sp* (31%), which represents the highest prevalence of parasitic infections detected among all the children included (Fig. 1).

Polyparasitism carrying two to three different species were detected in 19.7% (12/61) of infected children. Ten of the polyinfected children harbored two parasitic species (83.3%) and two of them harbored three parasitic species (16.7%). The most representative polyinfection was that of *Encephalitozoon intestinalis* and *Hymenolepis sp* with a prevalence of 41.7%, followed by that of *Cryptosporidium hominis/parvum* and *Hymenolepis sp* (25%). Polyinfections of *Cryptosporidium hominis/parvum/Encephalitozoon intestinalis* and *Hymenolepis sp/Trichuris trichiura* were detected in infected children with a prevalence of 16.7%, while polyinfections were poorly represented with a prevalence of 8.3%.

### Distribution of intestinal parasite infections by age and gender

In order to assess the distribution of intestinal parasites according to age, the children were subdivided into seven age groups ranging from 2 to 169 months. The overall prevalence of intestinal parasites (85.2%; *n* = 52/61) was found in children under 49 months of age. Children under 6 months of age were infected with 3 parasitic species which are *Encephalitozoon intestinalis, Hymenolepis sp,* and *Trichuris trichiura.* In the 6 to 24 months age group, five species were found (*Dientamoebla fragilis, Encephalitozoon intestinalis, Cryptosporidium hominis/parvum, Trichuris trichiura* and *Hymenolepis sp*). In the 25 to 48 months age group, *Enterocytozoon bieneusi, Encephalitozoon intestinalis, Cryptosporidium hominis/parvum and Hymenolepis sp* were identified (Table 3). Otherwise, in the 49 to 72 months age group, only *Hymenolepis sp* was detected. The 73 to 96 months, 97 to 120 months and the over 121 months age groups were infected with two different parasite species. The prevalence of intestinal parasites was statistically significant among the age groups (*p* = 0.03). The global prevalence of intestinal parasites was higher in the 6 to 24 months age group compared to the less than 6 months age group (p = 0.03). Similarly, a statistically significant difference in prevalence was also observed between the 6 to 24 months age group and the 49 to 72 months (*p* = 0.02), 73 to 96 months (p = 0.03), 97 to 120 months (p = 0.03) and the over 120 months (p = 0.03) age groups.

**Table 3:** Distribution of intestinal parasites by age groups of children

Type of parasite	< 6 (n = 4)	6–24 (*n* = 40)	25–48 (n = 8)	49–72 (*n* = 3)	73–96 (n = 2)	97–120 (n = 2)	≥ 121 (*n* = 2)	N° (%)
	N° (%)	N° (%)	N° (%)	N° (%)	N° (%)	N° (%)	N° (%)	
*Enterocytozoon bieneusi*	0 (0)	0 (0)	0 (0)	0 (0)	0 (0)	0 (0)	1 (50)	1 (1.6)
*Dientamoeba fragilis*	0 (0)	4 (10)	0 (0)	0 (0)	0 (0)	0 (0)	0 (0)	4 (6.6)
*Encephalitozoon intestinalis*	1 (25)	6 (15)	1 (12.5)	0 (0)	0 (0)	0 (0)	0 (0)	8 (13.1)
*Cryptosporidium hominis/parvum*	0 (0)	7 (17.5)	3 (37.5)	0 (0)	1 (50)	1 (50)	1 (50)	13 (21.3)
*Hymenolepis sp*	2 (50)	15 (37.5)	1 (12.5)	3 (100)	1 (50)	0 (0)	0 (0)	22 (36.1)
*Trichuris trichiura*	0 (0)	1 (2.5)	0 (0)	0 (0)	0 (0)	0 (0)	0 (0)	1 (1.6)
*Encephalitozoon intestinalis/Hymenolepis sp*	1 (25)	2 (5)	1 (12.5)	0 (0)	0 (0)	0 (0)	0 (0)	4 (6.6)
*C. hominis/parvum/Hymenolepis sp*	0 (0)	1 (2.5)	1 (12.5)	0 (0)	0 (0)	0 (0)	0 (0)	2 (3.3)
*C. hominis/parvum/Encephalitozoon intestinalis*	0 (0)	2 (5)	0 (0)	0 (0)	0 (0)	0 (0)	0 (0)	2 (3.3)
*Trichuris trichiura/Hymenolepis sp*	0 (0)	1 (2.5)	0 (0)	0 (0)	0 (0)	0 (0)	0 (0)	1 (1.6)
*Trichuris trichiura/Cryptos. hominis/parvum*	0 (0)	0 (0)	0 (0)	0 (0)	0 (0)	1 (50)	0 (0)	1 (1.6)
*Encephalitozoon intestinalis/Trichurus trichiura/Hymenolepis sp*	0 (0)	1 (2.5)	0 (0)	0 (0)	0 (0)	0 (0)	0 (0)	1 (1.6)
*C. hominis/parvum/Enterocytozoon bieneusi/Hymenolepis sp*	0 (0)	0 (0)	1 (12.5)	0 (0)	0 (0)	0 (0)	0 (0)	1 (1.6)

The prevalence of intestinal parasites by gender among infected children was assessed. Although the difference in the prevalence of intestinal parasites was not statistically significant between males and females, boys had a higher prevalence (63.3%) than girls 57.5%). Among the six parasitic species detected, *T. trichiura* and *Enterocytozoon bieneusi* were present only in the male population. However, the other four remaining species were found in both male and female children (data not shown).

### Distribution of intestinal parasites species by season

**Fig. 2:**
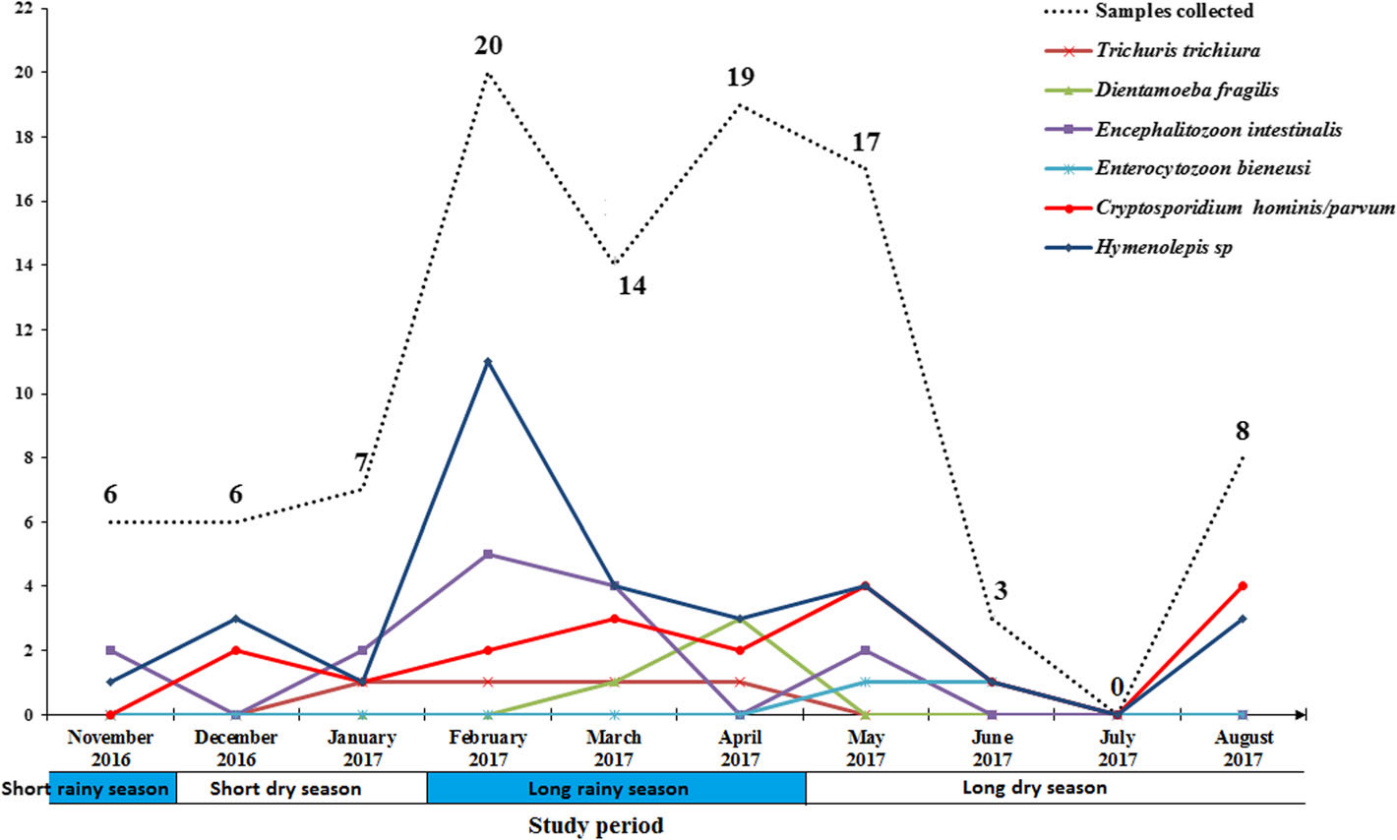
Seasonal distribution of intestinal parasites in children. Each point represents the number of positive cases of each parasite

The prevalence of parasites was unevenly distributed from month to month over a 1 year period (Fig. 2). The highest prevalence of parasitic infections was observed during the short dry season (December to January) and the long dry season (May to September), (76.9%: *n* = 10/13 and 64.3%: *n* = 18/28, for the short dry season and the long dry season, respectively). Moreover, the lowest prevalence of parasitic infections was seen during the months of February to April (long rainy season) and in November (during the short rainy season), (58.5%: *n* = 31/53 and 33.3%: *n* = 2/6, for the long rainy season and the month of November, respectively). The distribution of intestinal parasites was also analyzed monthly. The distribution of *Hymenolepis sp* was consistently found throughout the year except in July with the highest prevalence observed in February which corresponds to the long rainy season (35.5%: *n* = 11/31). The same is true for *Encephalitozoon intestinalis*, *Cryptosporidium hominis/parvum* and *Trichuris trichiura* and *Cryptosporidium hominis/parvum* whose prevalence peaked during the long rainy season and the month of May (long dry season). For *Dientamoeba fragilis*, the highest prevalence was observed in April and *Enterocytozoon bieneusi* was found in May and June only. The distribution of parasite species by month was significantly different (*p* = 0.0003).

### Association between intestinal parasites and age groups

**Fig. 3:**
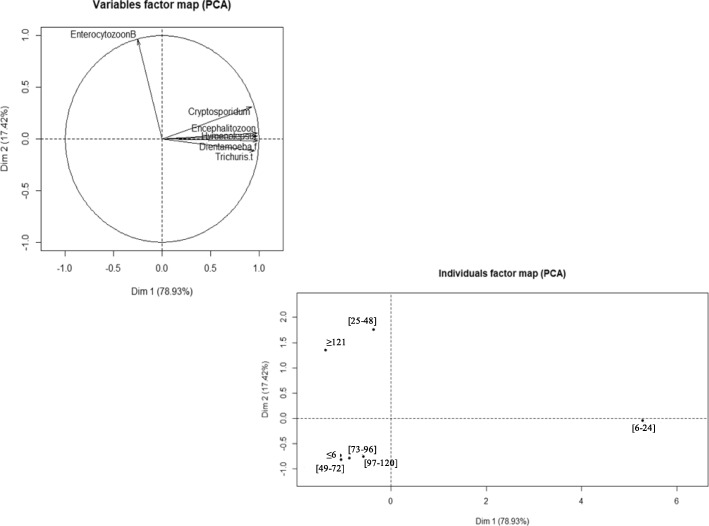
Correlation between the different parasites and distribution by age group. The points on individuals factor map (PCA) represent the different clinical groups

The association between the different parasitic species and the distribution according to the different age groups was determined using the principal component analysis (PCA). The results of the correlation analysis showed that the main parasitic species including *Cryptosporidium hominis/parvum, Encephalitozoon intestinalis, Hymenolepis sp, Dientamoeba fragilis* and *Trichuris trichiura* were positively correlated with each other. Only *Enterocytozoon bieneusi* was negatively correlated with four other species including *Encephalitozoon intestinalis, Hymenolepis sp, Dientamoeba fragilis* and *Trichuris trichiura* (r = − 0.4, − 0.3 and − 0.1, respectively)*.* No correlation was found between *Enterocytozoon bieneusi* and *Cryptosporidium hominis/parvum*. In addition, the superposition of the correlation circle with the distribution of parasites according to age groups showed that five parasitic species, including *Cryptosporidium hominis/parvum, Encephalitozoon intestinalis, Hymenolepis sp, Dientamoeba fragilis* and *Trichuris trichiura* were mainly associated in children aged 6 to 24 months. *Enterocytozoon bieneusi* was associated in children 25 to 48 months old and those older than 121 months (Fig. 3).

## Discussion

This study on diarrheal disease in Gabonese children is the first to assess the prevalence of intestinal parasite infections in Franceville, Gabon. While data on adults and children already exist, children with diarrheal syndrome living in the urban environment of the city of Franceville have not previously been studied.

In this present study, we showed that 88% of the children with diarrhea were under the age of five. Our results are similar to those of a previous study performed in Franceville between 2010 and 2011 reporting a high prevalence of diarrhea in children aged one to 5 years old [20]. Similarly, in Yaoundé, Cameroon, a prevalence of 78% was also found in 2005 among children under 5 years old and living in disadvantaged environments. This study reported a prevalence of 21.5% among children of the same age group and living in residential neighborhoods [23]. Another study conducted in Ethiopia in 2017 reported a prevalence of acute diarrhea of 11.9% among children under five in slums of Addis Ababa, which was significantly associated with inadequate sanitation and poor hygienic practices [24]. The results showing low diarrhea prevalence could be explained by recently implemented awareness programs aimed at improving sanitation and hygiene conditions in the areas studied. It is thus suggested that such programs be consistently implemented in order to curtail socioeconomic and behavioral factors that promote high prevalence of diarrhea in burdened countries.

In our study, creatinine levels are significantly higher in infected diarrheal children than in uninfected diarrheal children. Indeed, a previous study determined the proportion of morbidity attributed to *S. haematobium* infection and showed that, based on the results of prevalence ratios and attributable fractions, urine albumin-to-creatinine ratio (UACR) was identified as the most reliable tool for detecting schistosome-related morbidity, followed by dipsticks, visual urine inspection, questionnaires and finally, clinical examination. In addition, prevalence of albuminuria determined using UACR was positively associated with the presence of microhaematuria and proteinuria detected by dipsticks. Their finding suggests that these indicators used in combination can be a better predictor of the presence of urinary tract morbidity due to *S. haematobium* infection in children than the use of a single test parameter, which would thereby facilitate effective and timely interventions [25].

A range of different pathogenic organisms can cause pediatric diarrhea in the world, especially in tropical and developing countries, including rotaviruses and adenoviruses [20, 26] and bacteria such as *Shigella* and *Eschirichia coli* [27]. In the current study, we have shown that a significant percentage of pediatric diarrhea cases (61%) are also associated with the presence of intestinal parasitic infections. *Hymenolepis sp* was the most common pathogen accounting for 31% of cases. The second most frequent parasite in these children was *Cryptosporium hominis/parvum* with a prevalence of 19%. Our results are consistent with other studies showing that *Cryptosporium sp* was one of the pathogens associated with diarrhea [28, 29]. This parasite was also linked to diarrhea in children under 5 years old and was the second leading cause of diarrhea in children after rotavirus [27].

Molecular diagnostic results showed that protozoan infections were more common compared to STH infections (2 × 10^− 6^). The highest prevalence of protozoa has also been observed in African countries including Gabon [19, 30, 31]. The low prevalence of STH infections in our study could be explained by the systematic prescription of an antihelminthic in case of digestive symptoms. Studies in Gabon have shown a reduction in the prevalence of intestinal helminths since the introduction of antihelminthics. In a clinical trial conducted in Lambarene to evaluate the efficacy of single-dose versus repeated-dose of albendazole, a significant decrease in the prevalence of intestinal parasitosis was observed [32, 33]. However, the slow development of antiparasitic immunity in children and the lack of awareness regarding the importance of personal hygiene practices may explain the high prevalence of protozoa observed in our study. Furthermore, a study conducted in Cameroon in 2011, concluded that the prevalence of protozoan infections was significantly reduced from 50.9 to 25.9% as a result of health education [34]. Other authors, however, have observed a higher prevalence of helminths compared to protozoa. The most common helminth in our study was *Hymenolepis* with a prevalence of 31% which is similar to other studies conducted in Burkina-Faso [35].

The global prevalence of intestinal parasitic infection was 61%. Positivity was based on the results of molecular diagnostic, not on microscopic examination. However, a study conducted in different settlements of Gabon showed 61.1% of intestinal parasitic infections among school-aged children and adults by direct microscopic examination and the merthiolate-iodine-formaldehyde (thimerosal) concentration (MIFc) method [29]. Another study carried out in Lambaréné showed 21% of prevalence in school-age children after direct examination [17, 36]. Parasites were not seen in our study by direct examination. Our inability to use a concentration technique may have led to the underestimation of the frequency of certain parasites. We confined ourselves to results obtained from a single parasitological examination of the stool which may have underestimated the rates of microscopic intestinal parasitism in these children.

Polyparasitism was observed in our study population with a prevalence of 19.7%. In 12 polyparasitic children, 83.3% had double parasitism and 17.6% had triple parasitism. These results are consistent with those obtained in other African countries [37]. However, this rate is lower than that found in previous studies conducted in Gabon and other countries. In fact, a study conducted in different regions of Gabon showed that residing in a rural area represents a high risk factor for intestinal parasites infections [19]. Also, as seen in our study, the prevalence of IPIs observed in Gabon could be explained by the level of urbanization which remains very low in the country. Indeed, generally inadequate personal hygiene, unsafe water supplies, low levels of parental education and environmental conditions have been associated with polyparasitism.

High incidence of diarrhea were recorded between February (20%) and May (17%), which corresponds to the long rainy season. Other studies have also shown seasonality of rotavirus between the months of February and April [20]. High rainfall would contribute to the establishment of adequate wet conditions in which eggs and larvae could survive in the soil [38]. In terms of proportion of parasite distribution by age group, the prevalence of parasitic infections was higher among young children. Different factors such as behavioral factors and playful activities could explain this link between age and parasitic infections as described in different regions of the world [39, 40]. No difference in intestinal parasitic infections was observed between the sexes. The prevalence of infections between boys and girls was not different either. Also, both sexes are similarly exposed to parasite-prone environments such as contaminated soil and water.

The different parasite associations highlighted in this study revealed that five parasite species were positively correlated and this link was age-dependent. In our study, *Cryptosporidium hominis/parvum* was associated with microsporidia with a prevalence of 25% which is higher than what was reported in other countries [41].

## Conclusions

Intestinal parasitic infections with *Cryptosporiduim hominis/parvum, Encephalitozoon intestinalis* and *Hymenolepis sp*, were more prevalent in children in our study population. *Hymenolepis sp* and *Cryptosporium hominis/parvum* were shown to be the most frequent pediatric diarrheic infections. Moreover, the high prevalence of parasitic infections in this population of children indicates that the protozoa and helminths concerned are very common in the Franceville environment and its surroundings. Like the other main diarrhea causing agents, intestinal parasite infections are a major public health problem in Gabon. In order to better understand these parasites’ distribution among pediatric diarrheic children in the region, further studies targeting sociodemographic characteristics, behavioral and hygienic practice status are required. Such studies will enable the implementation of multiple intervention strategies for children, households and their environments to reduce intestinal parasitic infections.

## Data Availability

The datasets that were used for the analysis and the preparation of this manuscript are available from the corresponding author on reasonable request.

## Abbreviations

EDCTP: European & Developing Countries Clinical Trials Partnership
CANTAM: Central Africa Network on Tuberculosis, HIV/AIDS and Malaria
UNEEREP: Unité d’Evolution, d’Epidémiologie et de Résistances Parasitaires
